# The neural and cardiovascular effects of exposure of gram-positive bacterial inflammation in preterm fetal sheep

**DOI:** 10.1177/0271678X231197380

**Published:** 2023-10-12

**Authors:** Simerdeep K Dhillon, Christopher A Lear, Joanne O Davidson, Shoichi Magawa, Alistair J Gunn, Laura Bennet

**Affiliations:** Department of Physiology, 1415 The University of Auckland, Auckland, New Zealand

**Keywords:** Perinatal infection, gram-positive inflammation, neuro-inflammation, preterm brain injury, endothelial dysfunction

## Abstract

Perinatal infection or inflammation are associated with adverse neurodevelopmental effects and cardiovascular impairments in preterm infants. Most preclinical studies have examined the effects of gram-negative bacterial inflammation on the developing brain, although gram-positive bacterial infections are a major contributor to adverse outcomes. Killed Su-strain group 3 A streptococcus pyogenes (Picibanil, OK-432) is being used for pleurodesis in fetal hydrothorax/chylothorax. We therefore examined the neural and cardiovascular effects of clinically relevant intra-plural infusions of Picibanil. Chronically instrumented preterm (0.7 gestation) fetal sheep received an intra-pleural injection of low-dose (0.1 mg, n = 8) or high-dose (1 mg, n = 8) Picibanil or saline-vehicle (n = 8). Fetal brains were collected for histology one-week after injection. Picibanil exposure was associated with sustained diffuse white matter inflammation and loss of immature and mature oligodendrocytes and subcortical neurons, and associated loss of EEG power. These neural effects were not dose-dependent. Picibanil was also associated with acute changes in heart rate and attenuation of the maturational increase in mean arterial pressure. Even a single exposure to a low-dose gram-positive bacterial-mediated inflammation during the antenatal period is associated with prolonged changes in vascular and neural function.

## Introduction

Preterm-born infants have high rates of long-term neurodevelopmental disabilities.^1^ Brain injury and neurological impairments in preterm infants are strongly associated with intrauterine and postnatal infection/inflammation.^2,3^ For example, a multicentre prospective study in a cohort of 805 extremely preterm-born infants reported that mild to moderate grade fetal inflammatory response was associated with an increased risk of cerebral palsy (odds ratio 1.50, 95% CI 1.04–2.18).^4^ Preterm infants with chorioamnionitis also have cardiovascular and haemodynamic impairments.^5^

Studies in neonatal rats and fetal sheep have shown that perinatal systemic inflammation is associated with neuroinflammation, white matter damage and neuronal loss, as well as hypotension with generalised vasodilation and altered neurovascular coupling.^6–9^ However, the majority of these studies used a cell wall component of gram-negative bacteria (lipopolysaccharide; LPS) to induce inflammation. Surprisingly, there have been few studies of gram-positive bacteria such as group B streptococcus (GBS), which are a major contributor in many countries to adverse maternal and neonatal outcomes.^10,11^ Gram-positive and gram-negative bacteria have distinct cell wall structures, pattern-recognising receptors, and downstream signalling pathways, so they result in differential systemic and neuropathological effects.^12^ In neonatal mice (P8), exposure to intraperitoneal injections of TLR 4 agonist (LPS) or TLR 2 agonist (Pam3CSK4) was associated with a differential chemokine response in choroid plexus and the brain, and only Pam3CSK4 was associated with an increase in blood-brain barrier permeability and leukocyte infiltration.^13^

Importantly, a sclerosant agent derived from the low virulence Su-strain group 3 A streptococcus pyogenes (Picibanil (OK-432)) has been tested for inducing pleurodesis to treat re-occurring primary fetal hydrothorax.^14^ The inflammation induced by intra-pleural injection of Picibanil leads to formation of connective tissue between the parietal and visceral pleura, preventing the re-occurrence of fetal pleural effusions.^15^ The effect of this treatment on the developing brain has not been extensively evaluated. In 0.7 gestation fetal sheep, an intra-pleural injection of 0.1 mg Picibanil was associated with an acute suppression of EEG, body and breathing movements, progressive vasodilation in central and peripheral beds and one fetus developed bilateral hippocampal infarcts one week after Picibanil injection.^16,17^ These studies did not extensively assess neural injury and only examined the effects of a low dose of Picibanil (0.1 mg), whereas clinically, doses ranging from a single injection of 0.1 mg to multiple injections of 1 mg have been tested for fetal hydrothorax treatment.^14,18^ In this study, we compared the cardiovascular and neural effects of exposure to a low (0.1 mg) or high (1 mg) dose of Picibanil in 0.7 gestation fetal sheep.

## Methods

### Ethical approval

All procedures were approved by the Animal Ethics Committee of the University of Auckland (approval R1942) following the New Zealand Animal Welfare Act 1999, and carried out in accordance with the code of Animal ethical conduct established by the Ministry of Primary industries of New Zealand for the use of animals for teaching and research. All procedures comply with the ARRIVE guidelines.

### Fetal surgery

Twenty-four Romney-Suffolk cross fetal sheep were sourced from the University farm and instrumented on gestation days 99 – 100 (term is ∼ 147 days). For twin pregnancies, only one fetus was surgically instrumented. Ewes were acclimatised to laboratory conditions for one week before the surgery, during which time, regular veterinary and welfare checks were performed. Food, but not water, was withdrawn 12–18 hours before the surgery to reduce the risk of aspiration. Ewes were given an intramuscular injection of the antibiotic oxytetracycline (20 mg/Kg Phoenix Pharm, Auckland, New Zealand) 30 minutes before surgery for prophylaxis. Anaesthesia was induced by an intravenous (*i.v.*) injection of propofol (5 mg/kg, AstraZeneca, Auckland, New Zealand), and maintained with 2 to 3% isoflurane in oxygen after intubation. The depth of anaesthesia, maternal respiration and heart rate was constantly monitored during surgery by trained anaesthetic staff. Maternal fluid balance was maintained by a continuous *i.v.* infusion of Plasma-Lyte 148 (∼250 ml/h) (Baxter, Auckland, New Zealand).

All surgical procedures were performed using aseptic techniques, as previously described.^17,19^ A maternal midline incision was made to expose the uterus, and the fetus was partially exteriorised for instrumentation. Left femoral and right brachial arteries were catheterised with saline-filled polyvinyl catheters (SteriHealth, Dandenong South, VIC, Australia) for blood pressure measurement and pre-ductal fetal blood sampling, respectively. An additional catheter was secured to the fetus to measure amniotic fluid pressure. A saline-filled silicone catheter (2.16 mm OD, 1.02 mm ID, Bamford Ltd., Lower Hutt, New Zealand) was placed in the pleural space at the level of the seventh intercostal space, 1.5 cm lateral to sternum at the depth of 4 cm for administration of Picibanil.

An ultrasound flow probe (3S type, Transonic Systems Inc., New York, USA) was placed around a carotid and femoral artery to measure blood flows. Carotid blood flow was used an index for global cerebral perfusion.^20^ A pair of electrodes (AS633-3SSF wire, Cooner Wire, Chatsworth, CA, USA) were placed subcutaneously across the fetal chest to measure fetal electrocardiogram (ECG). Two pairs of electrodes (AS633-7SSF, Cooner Wire) were placed through burr holes on the dura over the parietal parasagittal cortex bilaterally, 5 and 10 mm anterior, 5 mm lateral to the bregma to measure fetal electroencephalographic (EEG) activity. The burr holes were sealed with surgical wax and electrodes fixed in place using cyanoacrylate glue. A reference electrode was placed over the occiput.

On completion of fetal instrumentation, fetal catheters were heparinised (20 U/ml heparin in saline), the fetus was returned to the uterus, and the amniotic fluid lost during surgery was replaced with sterile 0.9% saline (∼500 ml at 39 °C). The uterus was closed, and the antibiotic Gentamicin (80 mg, Pfizer, Auckland, New Zealand) was administered into the amniotic sac. All the fetal leads were exteriorised through the maternal flank. The maternal midline skin incision was repaired and infiltrated with a local analgesic: 10 ml 0.5% bupivacaine plus adrenaline (AstraZeneca Ltd., Auckland, New Zealand). A maternal long saphenous vein was catheterised for post-operative care.

Following surgery, animals were housed together in individual metabolic cages with access to food and water *ad libitum*. Rooms were temperature-controlled (16 ± 1 °C, humidity 50 ± 10%) with a 12:12 hour light:dark cycle. A period of 4–5 days of recovery was allowed before the commencement of experiments. Antibiotics were given *i.v.* to the ewe each day for 4 days; 600 mg benzyl-penicillin sodium (Novartis, Auckland, New Zealand) and 80 mg gentamicin (Pfizer, Auckland, New Zealand). Fetal and maternal vascular catheters were maintained patent by continuous infusion of heparinised saline (20 U/ml at a rate of 0.20 ml/hour).

### Physiological recordings

Fetal mean arterial blood pressure (MAP) (Novatrans II, MX860; Medex, Hilliard, OH, USA), corrected for maternal movement by subtraction of amniotic fluid pressure, fetal heart rate derived from the ECG, carotid blood flow (CaBF), femoral blood flow (FBF) and EEG were recorded continuously from 24 hours before until the end of experiments. Data was stored for offline analysis using custom data acquisition software (LabVIEW for Windows). Blood pressure signals were low-pass filtered with fifth-order Butterworth filter with a cutoff frequency at 20 Hz and then digitised at a sampling rate of 512 Hz. The raw ECG signal was filtered with an analogue first-order high-pass filter with a cutoff frequency set at 0.05 Hz and a fifth-order low-pass Bessel filter with a cutoff at 100 Hz and digitised at sampling rate of 1024 Hz. R-R intervals were extracted from this signal to calculate heart rate. CaBF and FBF signals were low-pass filtered with a second-order Butterworth filter at 0.1 Hz, digitised at 512 Hz.

The EEG signals were amplified 10,000 x fold and then processed with a first-order high-pass filter at 1.6 Hz and an analogue fifth-order low-pass Butterworth filter with a cutoff frequency set at 500 Hz. The signal was then filtered by a low-pass filter with a digital IIR Type 2 Chebyshev filter with a cutoff frequency of 128 Hz and saved at 256 Hz for analysis of raw EEG waveforms for seizures. The real-time power spectra and associated parameters were extracted from four-second epochs. Total EEG power (in microvolts squared (µV^2^)) was calculated on the power spectrum between 1 and 20 Hz. EEG power was log-transformed (EEG amplitude (dB), 10 x log (power)) to give a better approximation of a normal distribution. Spectral edge frequency was calculated as the frequency below which 90% of the power was present. The nuchal EMG signal was amplified 4000 × fold and filtered with a sixth-order low-pass Butterworth filter, with a cut-off frequency of 2 kHz. Signals were band-pass filtered between 100 and 1000 Hz and integrated using a time constant of 0.1 seconds and digitised at 512 Hz.

### Experimental design

Experiments were conducted at 104–105 (104.8 ± 0.4) days of gestation. At this age, brain maturation of fetal sheep is broadly equivalent to preterm human infants of 28 to 32 weeks of gestation.^21^ Fetuses were randomly assigned to the following groups: Saline-vehicle (Sal-Veh n = 8), Low-dose Picibanil (LD Pici, n = 8), or High-dose Picibanil (HD Pici, n = 8). Fetuses received either an intra-pleural infusion of saline, or low-dose (0.1 mg, 1 K.E.) or high-dose (1 mg, 10 K.E.) Picibanil dissolved in 10 ml saline over 20 minutes. All the experiments were begun between 9:30am and 10am. Fetal arterial blood samples were taken at 1 hour prior to the infusions, and then 1, 2, 4, 6, 24, 48, 72, 96, 120, 144, and 168 hours after Picibanil infusion for pH and blood gas analysis (ABL800-Basic, Radiometer, Copenhagen, Denmark) and for glucose and lactate measurements (YSI model 2300, Yellow Springs, OH, USA).

Seven days after vehicle or Picibanil exposure, ewes and fetuses were killed by an intravenous overdose of pentobarbitone sodium (9 g) to the ewe (Pentobarb 300; Chemstock International, Christchurch, New Zealand). This method is consistent with the Animal Welfare Act of New Zealand. Fetal brains were perfusion fixed via cannulation of both carotid arteries with 500 ml isotonic heparinised saline (20 IU/ml) followed by 1 litre of 10% phosphate-buffered formalin (Global Science, Auckland, New Zealand). Fetal body and organ weights were measured.

### Immunohistochemistry

Brain tissue was post-fixed by immersion in 10% phosphate-buffered formalin for one week, then processed and embedded in paraffin. 10 µm coronal sections were cut at the level of the mid-striatum and dorsal hippocampus using a microtome (Leica Jung RM2035, Leica Microsystems Ltd., Albany, New Zealand), and mounted on poly-L-lysine (Sigma-Aldrich) coated slides and oven dried.

Immunohistochemical staining was performed as previously described.^19^ The following primary and secondary antibodies were used: rabbit anti-neuronal nuclei monoclonal antibody (NeuN, Ab177487, Abcam, Cambridge, England), mouse monoclonal anti-2′3′-cyclic nucleotide 3′-phosphodiesterase (CNPase, Ab6319, Abcam), rabbit monoclonal anti-oligodendrocyte transcription factor-2 antibody (Olig-2, Ab109186, Abcam), polyclonal rabbit anti-ionised calcium binding adaptor molecule 1 (Iba1, Ab178680, Abcam), Rabbit polyclonal anti-cleaved caspase-3 (9579S, Cell Signaling Technology), anti-mouse biotinylated monoclonal IgG (BA-9200, Vector Laboratories, Burlingame, California, USA, 1:200) or anti-rabbit biotinylated monoclonal IgG (BA-1000, Vector Laboratories, 1:200). Sections taken 23 mm from stereotaxic zero were used to analyse white matter tracts in the periventricular region and intragyral tracts in parasagittal regions and striatum (including caudate nucleus and putamen). The cornu ammonis (CA) of the dorsal horn of the anterior hippocampus (divided into CA1/2, CA3, CA4, and dentate gyrus) was analysed using sections taken 17 mm anterior to stereotaxic zero.

For quantification of surviving neurons (NeuN), immature and mature oligodendrocytes (CNPase), the total number of oligodendrocytes (Olig2), cleaved caspase-3 positive apoptotic cells, and microglia and macrophages (amoeboid and ramified) (Iba1), regions of interest were imaged at 20 × magnification (0.2 µm/pixel) using light microscopy with a field size of 2560 × 1920 pixels on a Nikon 80i microscope equipped with a DS-Fi1-U3 camera and NIS Elements Br 4.0 imaging software (Nikon Instruments, Melville, New York, USA). In total, three fields in white matter tracts, four fields in the striatum (two in caudate nucleus and two in putamen) and one field in each subdivision of hippocampus were imaged in each hemisphere. Numbers of positively stained cells were quantified by manual counts using ImageJ software (National Institutes of Health, USA). The average cell count in each region from both hemispheres was calculated.

### Data analysis

All physiological and histological data were analysed by a single assessor (SD) blinded to the treatment protocols by using computer-generated codes for the experimental groups. Offline analysis of fetal physiological parameters was performed with customised LabVIEW programs. Data were assessed as hourly averages, with the 24 hours before the experiment used as the baseline period. EEG power was normalised to baseline. Stereotypic evolving seizure activity was visually identified on the raw EEG recording and defined as the appearance of sudden, repetitive, evolving stereotypic waveforms, lasting more than 10 seconds with an amplitude greater than 20 µV.^22^ Femoral vascular conductance (FVC) was calculated as blood flow/mean arterial pressure.

Statistical analysis was performed using SPSS version 28 (SPSS Inc., Chicago, IL, USA). For the continuously recorded physiological parameters and immunohistochemical data, between group comparisons were performed by two-factor mixed-design analysis of variance (ANOVA), with time or regions of interest as repeated measures and groups (saline control, low and high dose Picibanil) as independent variables. Post-hoc tests were only performed when a significant overall effect of group or interaction between group and time was found. Between group comparisons were performed by univariate analysis with Tukey’s post-hoc test. For fetal biochemical parameters, between group comparisons were made with one-way ANOVA with groups as an independent variable, followed by Tukey’s post-hoc test. Statistical significance was accepted as P < 0.05. All data are presented as mean ± SD.

## Results

### Baseline period, fetal blood biochemistry and post-mortem findings

There were no differences in fetal physiology and arterial blood biochemistry between the groups during the baseline period and no differences in the sex distribution between groups (female: male ratio Sal-Veh: 4:4, LD Pici 3:5, HD Pici 4:4). There were no changes in fetal blood biochemistry after Picibanil exposure (Table 1). At post-mortem, body and brain weights were not different between groups, but an increase in liver and spleen weights was observed in the low-dose Picibanil group (liver: P = 0.015, LD Pici vs. Sal-Veh; spleen: P = 0.015, LD Pici vs. Sal-Veh). In a subset of fetuses in the low-dose (5/8) and high-dose (3/8) Picibanil groups, adhesions were observed around the tip of the pleural catheter.

**Table 1.** Fetal biochemical parameters.

	Baseline	1 hour	2 hours	4 hours	6 hours	1 day	2 days	3 days	4 days	5 days	6 days	7 days
pH												
Saline-vehicle	7.37 ± 0.02	7.36 ± 0.01	7.37 ± 0.01	7.37 ± 0.02	7.37 ± 0.02	7.36 ± 0.02	7.35 ± 0.03	7.36 ± 0.02	7.36 ± 0.02	7.36 ± 0.02	7.36 ± 0.03	7.37 ± 0.05
Low-dose Picibanil	7.36 ± 0.02	7.37 ± 0.01	7.38 ± 0.01	7.41 ± 0.07	7.37 ± 0.02	7.39 ± 0.04	7.37 ± 0.01	7.35 ± 0.01	7.35 ± 0.02	7.34 ± 0.01	7.36 ± 0.01	7.35 ± 0.02
High-dose Picibanil	7.35 ± 0.03	7.35 ± 0.03	7.36 ± 0.03	7.36 ± 0.04	7.36 ± 0.03	7.36 ± 0.04	7.36 ± 0.03	7.35 ± 0.03	7.36 ± 0.02	7.36 ± 0.03	7.33 ± 0.06	7.33 ± 0.06
pO2 (mmHg)												
Saline-vehicle	26.4 ± 2.3	25.2 ± 2.9	25.0 ± 3.0	25.3 ± 3.2	24.9 ± 2.3	24.9 ± 2.3	25.3 ± 2.6	25.9 ± 4.2	25.5 ± 3.9	25.1 ± 1.9	25.2 ± 3.3	26.6 ± 3.3
Low-dose Picibanil	25.0 ± 2.8	25.0 ± 3.6	25.7 ± 2.8	25.2 ± 2.6	24.5 ± 2.3	22.7 ± 2.6	25.1 ± 3.5	25.1 ± 3.5	25.1 ± 2.8	25.3 ± 3.8	25.1 ± 3.3	24.0 ± 1.9
High-dose Picibanil	25.9 ± 2.0	27.1 ± 1.5	26.9 ± 1.7	26.4 ± 1.6	25.0 ± 2.3	23.8 ± 2.5	26.8 ± 2.9	25.2 ± 3.7	26.6 ± 1.6	25.5 ± 1.8	27.3 ± 4.0	23.4 ± 2.7
pCO2 (mmHg)												
Saline-vehicle	48.4 ± 4.6	46.3 ± 5.3	48.9 ± 3.5	48.7 ± 3.3	47.9 ± 4.3	50.8 ± 3.6	50.0 ± 3.4	50.7 ± 3.4	47.9 ± 6.3	49.5 ± 6.0	49.4 ± 4.0	51.2 ± 4.9
Low-dose Picibanil	48.8 ± 3.3	45.3 ± 3.6	46.1 ± 2.7	44.4 ± 3.8	47.6 ± 2.9	46.9 ± 3.0	47.3 ± 2.5	46.5 ± 5.7	48.5 ± 2.6	48.1 ± 2.3	46.0 ± 3.0	49.1 ± 2.9
High-dose Picibanil	49.0 ± 2.2	45.0 ± 2.7	46.5 ± 3.6	45.8 ± 2.7	50.3 ± 2.0	49.8 ± 2.0	49.1 ± 1.6	50.6 ± 1.6	51.2 ± 1.9	49.6 ± 3.5	50.3 ± 4.3	52.1 ± 1.1
Glucose (mmol/L)												
Saline-vehicle	1.0 ± 0.2	1.0 ± 0.2	1.0 ± 0.2	1.1 ± 0.2	1.0 ± 0.2	1.0 ± 0.2	1.1 ± 0.1	1.0 ± 0.2	1.1 ± 0.1	1.1 ± 0.1	1.1 ± 0.1	1.0 ± 0.1
Low-dose Picibanil	1.0 ± 0.2	1.2 ± 0.2	1.1 ± 0.1	1.1 ± 0.1	1.1 ± 0.1	1.0 ± 0.2	1.1 ± 0.1	1.1 ± 0.2	1.1 ± 0.1	1.1 ± 0.1	1.0 ± 0.2	1.1 ± 0.1
High-dose Picibanil	1.0 ± 0.3	1.0 ± 0.3	1.1 ± 0.2	1.1 ± 0.3	1.1 ± 0.2	1.0 ± 0.3	1.0 ± 0.2	1.1 ± 0.2	0.9 ± 0.2	1.1 ± 0.3	1.0 ± 0.2	0.9 ± 0.1
Lactate (mmol/L)												
Saline-vehicle	0.8 ± 0.2	0.7 ± 0.1	0.8 ± 0.1	0.8 ± 0.2	0.9 ± 0.2	0.9 ± 0.1	0.8 ± 0.1	0.8 ± 0.1	0.8 ± 0.1	0.8 ± 0.1	0.9 ± 0.2	0.8 ± 0.2
Low-dose Picibanil	0.7 ± 0.1	0.8 ± 0.2	0.9 ± 0.2	0.9 ± 0.1	1.1 ± 0.2	0.8 ± 0.2	0.8 ± 0.2	0.8 ± 0.3	0.8 ± 0.1	0.8 ± 0.1	0.8 ± 0.1	0.9 ± 0.2
High-dose Picibanil	0.8 ± 0.2	0.8 ± 0.2	0.9 ± 0.2	1.0 ± 0.2	1.1 ± 0.3	0.8 ± 0.1	0.8 ± 0.1	0.7 ± 0.1	0.7 ± 0.0	0.8 ± 0.1	0.8 ± 0.2	0.9 ± 0.1
Haematocrit (%)												
Saline-vehicle	25.4 ± 0.8	24.8 ± 2.9	26.0 ± 2.6	25.8 ± 1.7	25.6 ± 2.0	26.5 ± 1.0	25.7 ± 3.0	25.9 ± 2.5	25.8 ± 3.5	26.1 ± 4.0	26.2 ± 4.0	26.8 ± 3.0
Low-dose Picibanil	26.4 ± 3.3	25.9 ± 3.6	26.9 ± 3.4	27.6 ± 4.3	28.5 ± 4.3	27.0 ± 3.7	28.0 ± 5.6	27.1 ± 5.1	27.7 ± 5.0	28.6 ± 4.1	27.3 ± 4.1	27.4 ± 4.7
High-dose Picibanil	26.2 ± 2.0	24.6 ± 2.4	25.3 ± 2.4	26.1 ± 2.3	28.4 ± 1.7	26.8 ± 2.3	26.8 ± 3.0	27.4 ± 3.5	27.0 ± 2.9	26.4 ± 2.6	26.5 ± 2.6	26.3 ± 2.0
SO_2_ (%)												
Saline-vehicle	71.2 ± 8.2	69.8 ± 9.6	66.7 ± 8.7	68.3 ± 8.7	70.1 ± 6.9	67.4 ± 6.5	70.2 ± 4.9	69.4 ± 5.7	70.8 ± 11.6	66.0 ± 10.2	65.6 ± 10.4	68.9 ± 8.3
Low-dose Picibanil	70.5 ± 7.0	70.2 ± 10.2	72.2 ± 7.1	71.6 ± 6.2	69.6 ± 7.2	63.1 ± 4.0	68.0 ± 5.4	67.1 ± 5.1	65.7 ± 3.9	65.9 ± 4.9	68.5 ± 4.0	63.3 ± 4.6
High-dose Picibanil	73.0 ± 3.3	73.9 ± 3.6	74.5 ± 2.3	72.8 ± 4.5	68.8 ± 6.3	68.4 ± 5.5	75.1 ± 4.0	69.7 ± 7.0	75.3 ± 3.5	69.5 ± 4.4	71.5 ± 6.5	69.2 ± 1.8

Fetal arterial blood pH, partial pressures of oxygen and carbon dioxide, glucose and lactate, haematocrit and oxygen saturation 1 hour before, 1, 2, 4, 6, 24, 72, 96, 120, 144 and 168 hours after Picibanil infusion in saline-vehicle (n = 8), low-dose Picibanil (n = 8) and high-dose Picibanil (n = 8) groups. Data were analysed using one-way ANOVA, followed by Tukey’s post hoc comparisons. Data are mean ± SD.

### Fetal heart rate and blood pressure

**Figure 1.**
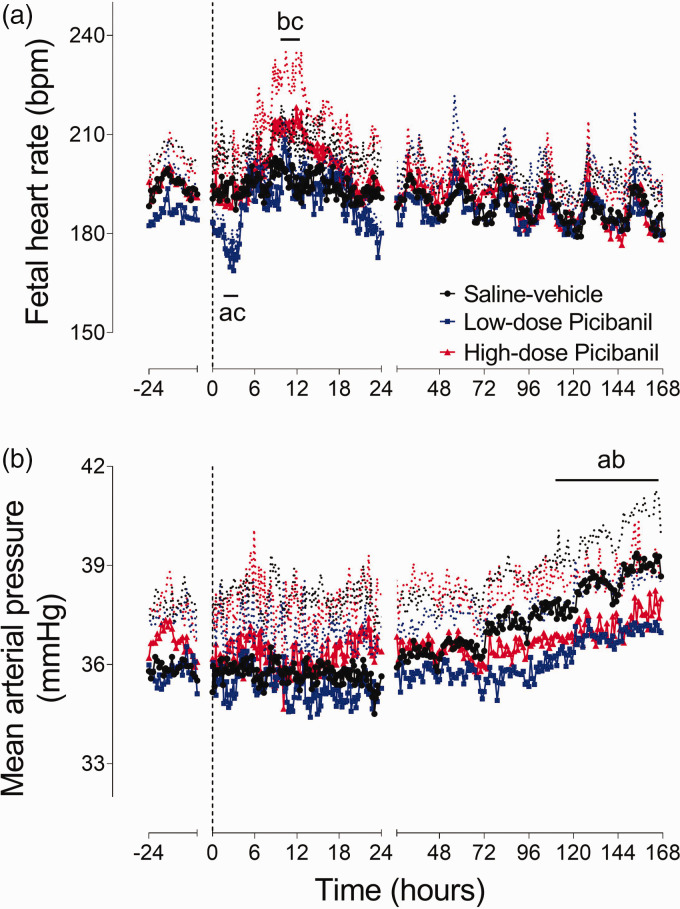
Time sequence of changes in fetal heart rate and mean arterial pressure. Changes in fetal heart rate (bpm, Panel a) and mean arterial pressure (mmHg, Panel b) for 24 hours before and 168 hours after intra-pleural infusions of saline or Picibanil in the saline-vehicle (n = 8, closed circles; black), low-dose Picibanil (n = 8, closed squares; blue) and high-dose Picibanil (n = 8, triangles; red) groups. The vertical dotted bar is the time when Picibanil was administered. Data are 10 min averages for the first 24 hours and hourly averages from 25–168 hours, presented as mean ± SD, and were analysed using mixed-design ANOVA with time as a repeated measure and experimental groups as independent factors. Between group comparisons were performed using Tukey’s post hoc test. Figure symbols are (a) saline-vehicle *vs.* low-dose Picibanil P < 0.05, (b) saline-vehicle *vs.* high-dose Picibanil P < 0.05 and (c) low-dose *vs.* high-dose Picibanil P < 0.05.

Fetal heart rate showed a significant interaction between group and time during the first 24 hours after Picibanil infusion (P = 0.021, Figure 1). The low-dose group showed a transient reduction in heart rate after Picibanil infusion, with the nadir at 3–4 hours (P = 0.001, Sal-Veh vs. LD Pici and P = 0.001, LD Pici vs. HD Pici). In contrast, there was no acute change in the high-dose group, but heart rate increased between 11 to 13 hours after Picibanil infusion (P = 0.017, Sal-Veh vs. HD Pici and P = 0.015, HD Pici vs. LD Pici).

There was no acute effect of Picibanil infusion on arterial blood pressure (Figure 1). Mean arterial pressure increased progressively with gestational age in the saline-vehicle group (35.8 ± .0.04 mmHg at baseline vs. 38.9 ± 0.05 mmHg on day 7, P = 0.030). Picibanil exposure was associated with a blunted maturational increase during the experimental period, and mean arterial blood pressure in the Picibanil groups was lower than the saline-vehicle group towards the end of recovery (P = 0.027, Sal-Veh vs HD Pici and P = 0.030, Sal-Veh vs LD Pici 125 to 168 h).

### Blood flow and vascular resistance

**Figure 2.**
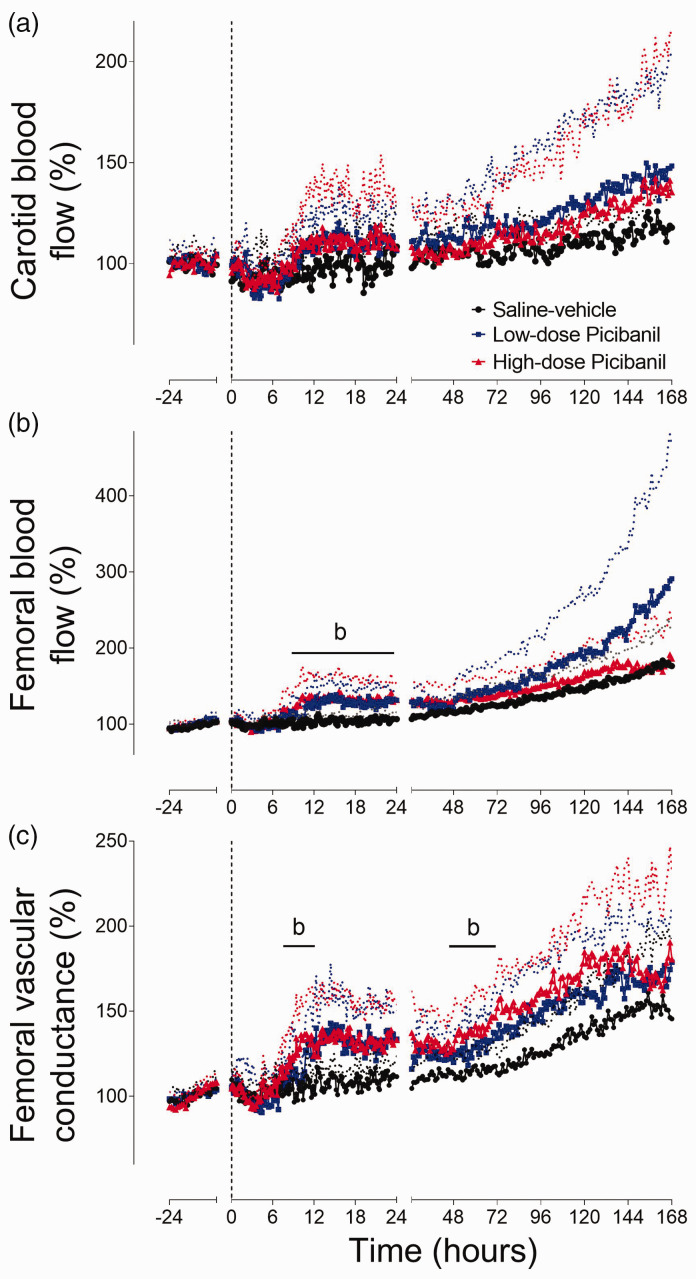
Time sequence of changes in blood flow and vascular resistance. Changes in carotid blood flow (%baseline, Panel a), femoral blood flow (%baseline, Panel b) and femoral vascular conductance (%baseline, Panel c) for 24 hours before and 168 hours after intrapleural Picibanil or saline infusion in the saline-vehicle (n = 8, closed circles; black), low-dose Picibanil (n = 5, closed squares; blue) and high-dose Picibanil (n = 6, triangles; red) groups. The vertical dotted bar is the time when Picibanil was administered. Data are 10 min averages for the first 24 hours and hourly averages from 25–168 hours, presented as mean ± SD, and were analysed using mixed-design ANOVA with time as a repeated measure and experimental groups as independent factors. Between group comparisons were performed using Tukey’s post hoc test. Figure symbols are (b) saline-vehicle *vs.* high-dose Picibanil P < 0.05.

Carotid artery blood flow (Figure 2) and vascular conductance (not shown) were not different between groups. Both the Picibanil groups showed a relative increase in femoral blood flow from 8 hours after the infusion. In the high-dose Picibanil group, femoral blood flow was significantly higher than saline-vehicle from 8 to 24 hours (P = 0.016, Sal-Veh vs HD Pici and P = 0.161, Sal-Veh vs LD Pici). This was associated with an increase in femoral vascular conductance in the high-dose Picibanil group (P = 0.019, Sal-Veh vs. HD Pici, P = 0.452, Sal-Veh vs. LD 8 to 12 h).

### Fetal body movements

**Figure 3.**
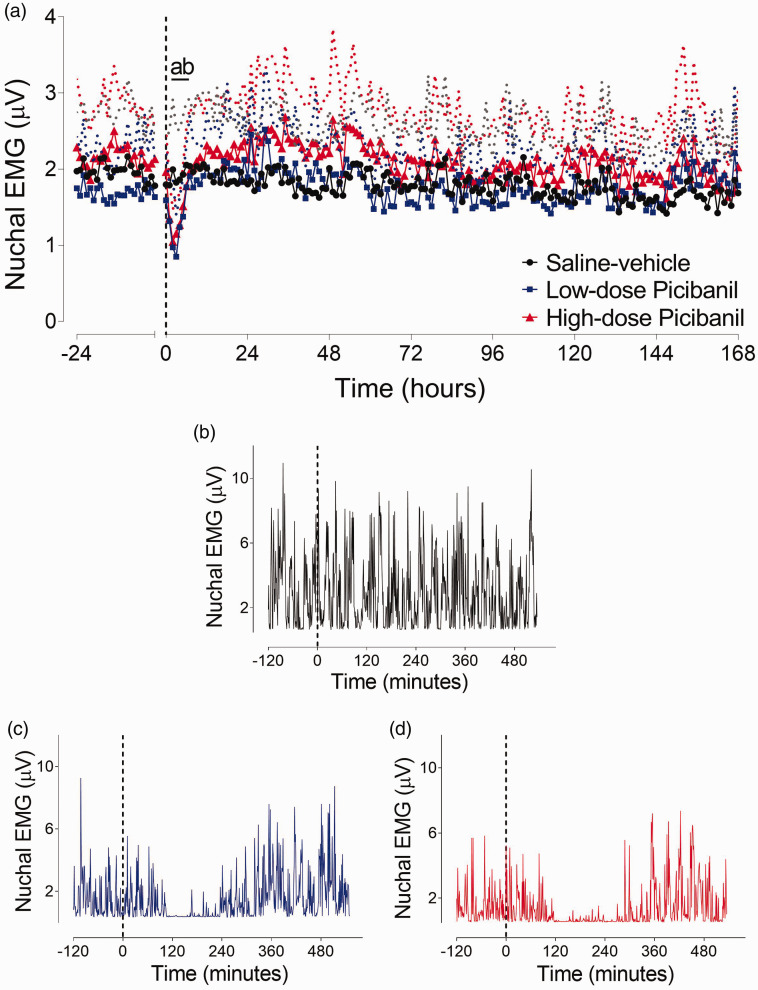
Time sequence of changes in fetal body movements. Changes in fetal body movements (µV) (Panel a) for 24 hours before and 168 hours after intra-pleural Picibanil or saline infusion in the saline-vehicle (n = 8, closed circles; black), low-dose Picibanil (n = 7, closed squares; blue) and high-dose Picibanil (n = 8, triangles; red) groups. The vertical dotted bars are the time when vehicle or Picibanil were infused. Data are hourly averages presented as mean ± SD, and were analysed using mixed-design ANOVA with time as a repeated measure and experimental groups as independent factors. Between group comparisons were performed using Tukey’s post hoc test. Figure symbols are (a) saline-vehicle vs. low-dose Picibanil P < 0.05 and (b) saline-vehicle vs. high-dose Picibanil P < 0.05. Examples of 1-minute average data of nuchal EMG (µV) during 2 hours before and 9 hours after the injection in the saline-vehicle (Panel b), low-dose Picibanil (Panel c) and high-dose Picibanil (Panel d) groups.

Body movements in the 0.7 gestation fetal sheep occurred nearly continuously without any specific sleep-state related pattern, unlike later in gestation^23^ (Figure 3). In both the Picibanil groups, fetal body movements were completely suppressed from 3 to 6 hours after the infusion (P = 0.019, Seh-Veh vs LD Pici and P = 0.014, Sal-Veh vs. HD Pici), with no significant difference between the Picibanil groups.

### EEG amplitude and spectral edge frequency

**Figure 4.**
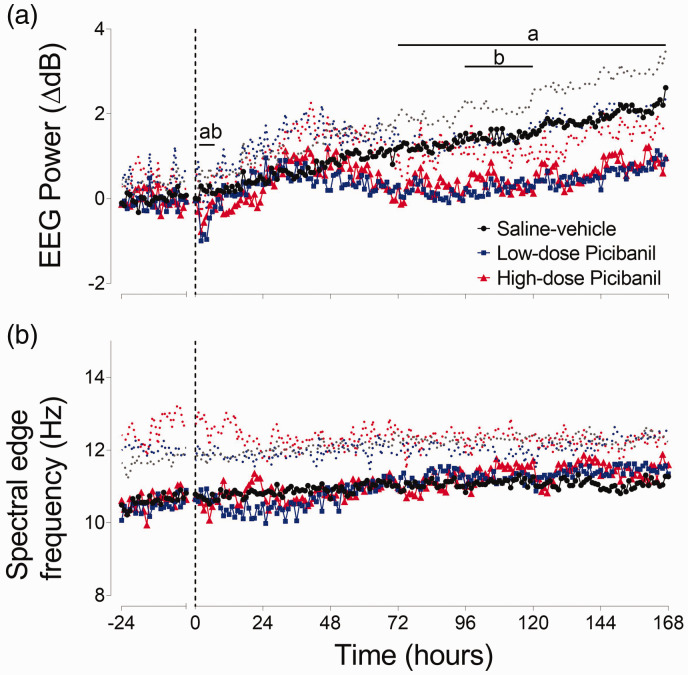
Neurophysiological recovery. Changes in EEG power (normalised to baseline, ΔdB, Panel a) and spectral edge frequency (Hz, Panel b) for 24 hours before and 168 hours after intra-pleural Picibanil or saline infusion in the saline-vehicle (n = 8, closed circles; black), low-dose Picibanil (n = 7, closed squares; blue) and high-dose Picibanil (n = 6, triangles; red) groups. The vertical dotted bars are the times when Picibanil was infused. Data are hourly averages presented as mean ± SD, and were analysed using mixed-design ANOVA with time as a repeated measure and experimental groups as independent factors. Between group comparisons were performed using Tukey’s post hoc test. Figure symbols are (a) saline-vehicle vs. low-dose Picibanil P < 0.05 and (b) saline-vehicle vs. high-dose Picibanil P < 0.05.

High and low-dose Picibanil exposure were associated with an acute reduction in EEG power from 3 to 6 hours (P = 0.003, Sal-Veh vs. LD Pici and P = 0.016, Sal-Veh vs. HD Pici) (Figure 4). Thereafter, a maturational increase in EEG power was observed in all groups, but EEG power remained significantly lower in both Picibanil groups than the saline-vehicle group from 72–168 hours in the low-dose group (P = 0.024, Sal-Veh vs. LD Pici) and from 96–144 hours in the high-dose group (P = 0.036, Sal-Veh vs. HD Pici). There was no difference between the high-dose and low-dose Picibanil groups from 72 hours onwards. There was no significant difference in the spectral edge frequency (Figure 4) or the percentage of EEG power in delta, theta, alpha and beta spectral bands (data not shown) between the groups at any time period during the recovery.

### Seizures

During the baseline period, the raw EEG in all groups showed mixed amplitude and frequency activity characteristic of immature, preterm EEG activity. No seizure activity was observed in the saline-vehicle group at any time. Only one fetus in the low-dose Picibanil group developed seizures, and this fetus had bilateral hippocampal infarcts (seizure onset 10.5 h, total seizure burden 45 h, seizure count 42, mean seizure burden 25.9 s/h, mean amplitude 49.8 µV and mean duration 27.8 s). 5/8 fetuses in the high-dose Picibanil group developed abnormal EEG activity after Picibanil infusion, ranging from stereotypic evolving seizures to seizure-like rolling activity (seizure onset 48.1 ± 28.0 h, total seizure burden 56.7 ± 35.6 h, seizure count 28.0 ± 17.4, mean seizure burden 20.2 ± 9.9 s/h, mean amplitude 84.9 ± 64.5 µV and mean duration 40.7 ± 15.2 s). The background EEG activity was not suppressed between the periods of abnormal high amplitude and low frequency waves.

### White matter inflammation

**Figure 5.**
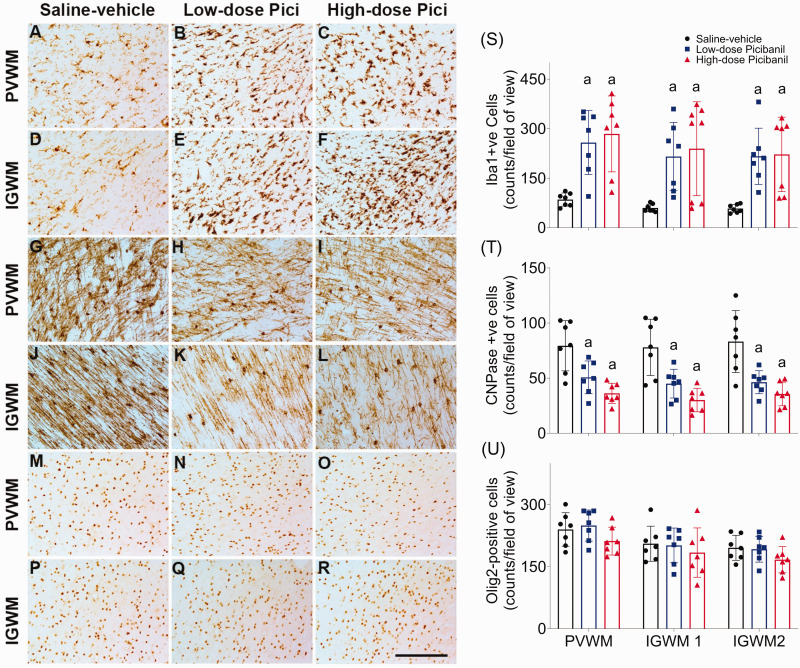
Photomicrographs showing microglia and macrophages (Iba1-postive cells) in the periventricular white matter (PVWM, Panels a–c) and intragyral white matter (IGWM, Panels d–f), immature and mature oligodendrocytes (2′,3′-Cyclic-nucleotide 3′-phosphodiesterase (CNPase)-positive cells) in PVWM, (Panel g–i), IGWM (Panels j–l) and total oligodendrocytes (Olig2 positive cells) in PVWM (Panel m–o) and IGWM (Panel p–r) and in the saline-vehicle, low-dose Picibanil and high-dose Picibanil groups. Scale bar is 50 µm. Cell density of microglia and macrophages (Iba1, panel s), immature and mature oligodendrocytes (CNPase, Panel t) and total oligodendrocytes (Olig2, panel u) in the periventricular and parasagittal intragyral white matter areas in the saline-vehicle (closed black circles, n = 7), low-dose Picibanil (closed blue squares, n = 7) and high-dose Picibanil (red triangles, n = 7) groups at one week after intra-pleural saline or Picibanil infusion. Data are presented as individual animals (the bars show mean and SD), and were analysed using mixed-design ANOVA with regions as repeated measures and experimental groups as independent variables. Comparisons between the groups were performed using Tukey’s post hoc test. Figure symbols: (a) P < 0.05 *vs.* saline-vehicle.

Picibanil infusion was associated with increased numbers of microglia and macrophages (Iba1-positive cells) in the PVWM (P = 0.005, Sal-Veh vs LD Pici; P = 0.001, Sal-Veh vs HD Pici), IGWM1 (P = 0.032, Sal-Veh vs LD Pici; P = 0.005, Sal-Veh vs HD Pici) and IGWM2 regions of the parasagittal gyrus (P = 0.006, Sal-Veh vs LD Pici; P = 0.002, Sal-Veh vs HD Pici, Figure 5). There were no significant differences between the low-dose and high-dose Picibanil groups.

### Oligodendrocyte survival

Picibanil infusion was associated with a loss of immature and mature oligodendrocytes (CNPase positive cells) in the PVWM (P = 0.012, Sal-Veh vs LD Pici; P = 0.001, Sal-Veh vs HD Pici), IGWM1 (P = 0.007, Sal-Veh vs LD Pici; P = 0.001, Sal-Veh vs HD Pici) and IGWM2 regions of the parasagittal gyrus (P = 0.004, Sal-Veh vs LD Pici; P = 0.001, Sal-Veh vs HD Pici, Figure 5). There was no significant effect of Picibanil exposure on total oligodendrocytes (Olig2 positive cells) or cleaved caspase-3 positive apoptotic cells (Supplementary Figure 1).

### Sub-cortical neuronal density

**Figure 6.**
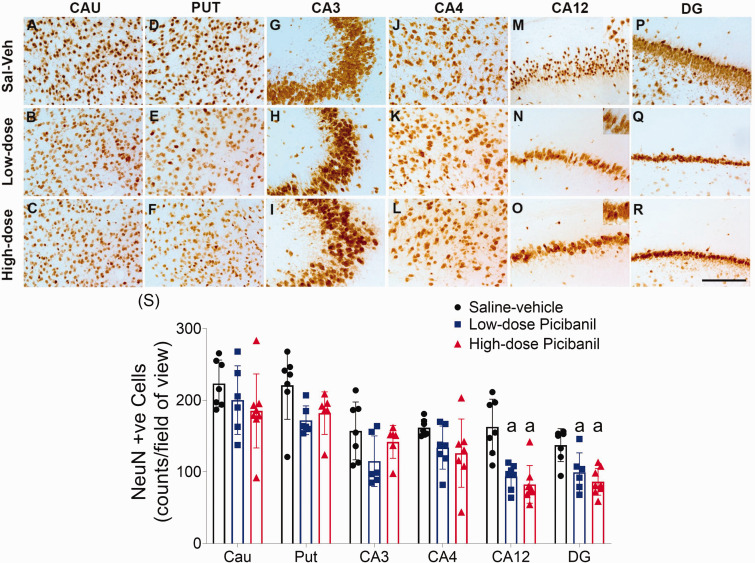
Photomicrographs of neurons (NeuN-positive cells) in the striatal caudate nucleus (caudate, panel a–c), putamen (Panels d–f), Cornu Ammonis (CA)3 (Panels g–i), CA4 (Panels j–l), CA12 (Panels m–o) and dentate gyrus (DG, panels p–r), of the hippocampus of saline-vehicle, low-dose Picibanil and high-dose Picibanil groups. Scale bar is 50 µm. Cell density of neurons (NeuN-positive) in caudate nucleus, putamen and CA3, CA4, CA12 and DG of the hippocampus in the periventricular and parasagittal intragyral white matter areas the saline-vehicle (closed black circles, n = 7), low-dose Picibanil (closed blue squares, n = 7) and high-dose Picibanil (red triangles, n = 7) groups at one week after intra-pleural Picibanil infusion (panel s). Data are presented as individual animals (the bars show the mean and SD), and were analysed using mixed-design ANOVA with regions as repeated measures and experimental groups as independent variables. Comparisons between the groups were performed using Tukey’s post hoc test. Figure symbols: (a) P < 0.05 *vs.* saline-vehicle. Cau: caudate nucleus, put: putamen, CA: cornu ammonis, DG: dentate gyrus.

Picibanil infusions were associated with significant neuronal loss in the CA1/2 (P = 0.013, Sal-Veh vs LD Pici; P = 0.002, Sal-Veh vs HD Pici), and DG regions of the hippocampus (P = 0.030, Sal-Veh vs LD Pici; P = 0.001, Sal-Veh vs HD Pici, Figure 6), with no significant difference between the Picibanil groups. There was no effect of Picibanil exposure on neuronal survival in the striatum or apoptotic cell death in the hippocampal sub-fields (Supplementary Figure 1). Qualitative assessment suggested that neurons in the CA12 region of the hippocampus in the Picibanil groups had a distinctive morphology, including an irregular structure and swollen cytoplasm, consistent with features of necrosis (Figure 6, Panel M-O).

## Discussion

The present study demonstrates that exposure to a single acute infusion of the gram-positive bacterial product Picibanil in preterm fetal sheep is associated with diffuse microgliosis and loss of immature and mature oligodendrocytes in the white matter tracts and hippocampal neurons after 7 days recovery. Intriguingly, the pattern and degree of histological injury were not dose-dependent, such that a similar attenuation of the maturational increase in EEG power was observed in the low (0.1 mg) and high (1 mg) dose Picibanil groups. Moreover, Picibanil exposure attenuated the maturational increase in mean arterial pressure in association with peripheral vasodilation, consistent with a sustained change in vascular endothelial function.

### Neuroinflammation and dose-independent neural injury

Consistent with previous reports,^16^ intra-pleural Picibanil infusion was associated with a local inflammatory response leading to pleural adhesions. Although we were not able to assess systemic inflammatory mediators in the present study, this likely resulted in a systemic inflammatory response, as pleural fluid is removed by bulk flow into the pleural lymphatic system.^24^ In turn, white matter inflammation was observed one week after both high and low-dose Picibanil, in association with similar loss of immature and mature oligodendrocytes. In the context of no change in total numbers of oligodendrocytes (Olig2+ve cells) after Picibanil, this finding infers that there was maturational arrest of oligodendrocytes.^25^ Microglial infiltration and neuroinflammation are a hallmark of diffuse white matter injury in the periventricular white matter in preterm human infants,^26^ and similar microglial activation and loss of mature oligodendrocytes has been observed in preterm fetal sheep at 10 to 15 days after systemic exposure to LPS.^27–29^

Picibanil infusion was also associated with a region-specific loss of hippocampal neurons, with injury to the CA1/2 and DG regions but no significant change in the CA3 and CA4 regions. Clinically, exposure to histological chorioamnionitis is associated with reduced hippocampal volume in preterm-born children.^30^ Studies in neonatal rats and mice have also shown that perinatal exposure to LPS-induced inflammation is associated with hippocampal neuronal loss and long-term structural and functional changes.^31,32^ The vulnerability of the hippocampus to inflammation may be related to its involvement in neuro-immune cross-talk due to proximity with the choroid plexus.^33^ Moreover, the hippocampus has a high density of interleukin receptors; in particular, neurons in sub-fields CA12 and DG are highly sensitive to pro-inflammatory cytokines,^34^ consistent with the present study.

Activated microglia play a pivotal role in mediating cell death, maturational arrest of neuronal precursors and oligodendrocyte progenitors, and altered synaptic function via increased production of pro-inflammatory cytokines and regulation of oxidative stress.^25,35^ In addition to the direct effect of neuroinflammation, potentially regional changes in oxygen delivery may contribute to neural injury.^36^ In the present study there was no change in global cerebral perfusion, but the effect of Picibanil on local microcirculation and tissue hypoxia was not assessed. Further, in neonatal rodents, impaired blood-brain barrier function associated with maternal-immune activation contributes to long-term changes in microglia.^37^ Thus, future studies should evaluate changes in blood-brain barrier and the neurovascular unit after Picibanil exposure.

The temporal profile of the evolution of cell death after exposure to gram-positive bacteria-mediated inflammation is not well characterised. In the present study, apoptotic cell death was not significantly upregulated in white matter or the hippocampus at one week after Picibanil exposure, and neurons in CA12 subfield of hippocampus showed features consistent with necrosis. We cannot rule out the possibility that apoptosis could have mediated early, acute cell loss. Alternatively, programmed necrosis, induced by the pro-inflammatory cytokine, tumour-necrosis factor (TNF) has been shown to be a key mediator of cell death in multiple neuro-inflammatory conditions.^38,39^

The absence of a dose-dependent response to Picibanil on the pattern and degree of neural injury in the present study suggests that the inflammation-inducing signalling pathways were similarly activated within the dose range examined in this study. The pattern of injury is similar to that previously reported after systemic exposure to other inflammatory stimuli such as LPS, *Ureaplasma Parvum* and *Candida Albicans* in preterm fetal sheep.^6,29,40,41^ These data collectively emphasise the role of sustained inflammation, induced by a range of different stimuli, in mediating neural injury in the preterm brain and highlights development-dependent regional vulnerability.

### Acute and long-term neurophysiological effects

Consistent with previous reports of acute neurophysiological effects of systemic inflammation in fetal sheep and adult rats,^7,16,42^ we observed a transient reduction of EEG power and inhibition of fetal body movements 3 to 6 hours after Picibanil injection, suggesting overall suppression of brain activity. This acute neural suppression was likely associated with induction of neuroinflammation during this period there was no cardiovascular compromise, hypoxia or cerebral hypoperfusion. Previous studies in fetal sheep have shown that systemic inflammation results in an acute upregulation of pro-inflammatory cytokines in blood and cerebrospinal fluid starting between 2–6 hours.^7,43^

After the initial transient EEG suppression, EEG power recovered to baseline, and the high-dose Picibanil group subsequently developed seizures, whereas in the low-dose group, only the fetus that developed hippocampal infarction developed seizures. Despite the absence of seizures in the low-dose group, there was no significant difference in neural injury between the groups. These findings support the concept that the seizure activity in the high-dose group reflects an acute functional abnormality secondary to a dose-dependent neuro-inflammatory response, but it did not contribute to the severity of neural injury after one week recovery. Pro-inflammatory cytokines such as IL-1β, TNF-α and IL-6 can reduce or increase neuronal excitability by altering pre-synaptic neurotransmitter release, modification of post-synaptic ion channels, changes in cerebral neurosteroid concentration and mitochondrial function.^43–45^ Such alterations to neuronal excitability may explain the appearance of seizure activity seemingly in absence of greater neural injury. Nonetheless, the reader should consider the possibility that the induction of seizure activity may have triggered injury or altered neural maturation in a manner not assessed in the present study, or which may only become evident at a later time-point.

Subsequently, Picibanil exposure was associated with a loss of maturational increase in EEG power. Similarly, subcortical grey and white matter damage after LPS exposure in preterm fetal sheep is also associated with reduced EEG amplitude.^7,46^ Consistent with these preclinical observations, in very preterm infants a decrease in EEG amplitude and absence of sleep-wake cycling during the first four days after birth was associated with exposure to perinatal inflammation.^47^ Further, sustained cerebral inflammation associated with Picibanil may also have modulated neuronal excitability and so impaired the maturational increase in EEG power. For example, in rat spinal cord cultures, prolonged treatment with IL-6 reduced voltage-evoked sodium currents and reduced action potential amplitude without affecting depolarisation-evoked activation.^48^

Interestingly, Picibanil did not alter the frequency of EEG activity. Maturational changes in EEG spectral frequency are associated with the development of thalamocortical, inter-neuronal and cortico-cortical connections.^47,49^ Thus, the lack of effect of Picibanil exposure on EEG frequency suggests that by one-week post-Picibanil infusion, overall synaptic development and communication between neural networks were not affected. However, given the presence of neural injury, with sustained inflammation and reduced EEG amplitude, we cannot exclude the possibility of longer term impairment of neuronal network development.

### Cardiovascular effects

Picibanil was associated with both acute and prolonged cardiovascular effects that differed as a function of dose. There was an acute reduction in heart rate after low-dose Picibanil exposure, which potentially reflects a direct chronotropic effect of inflammatory mediators.^50,51^ Alternatively it may represent activation of the parasympathetic system. In a subset of adult mice, an acute reduction in heart rate was observed after systemic injection of live gram-positive bacteria, which was inhibited by blockade of muscarinic cholinergic receptors.^52^ By contrast, in the high-dose group, there was no acute bradycardia, and a moderately elevated heart rate was observed from 11 to 15 hours. Afferent signalling to the brain via the cholinergic anti-inflammatory pathway can lead to recruitment of the HPA axis and sympathetic nervous system if there is sufficient immune activity.^53^ Further, sympathetic outflow can also be modulated by hypothalamic inflammation, and speculatively, greater sympathetic nervous system activity may have attenuated efferent parasympathetic activity and so contributed to an increase in heart rate in the high-dose group.

Picibanil exposure attenuated the maturational increase in mean arterial pressure. Lower mean arterial pressure was not associated with impaired fetal growth but was partly related to the change in vascular tone. Generalised vasodilation is commonly observed after inflammatory challenges.^54^ Consistent with this, we have previously reported that low-dose (0.1 mg) Picibanil infusion was associated with vasodilation in the peripheral and central beds.^17^ This response was not associated with an increase in plasma nitrite concentration, suggesting that altered responsiveness to vasoconstrictors may be more important in modulating the vascular tone in this setting. The attenuated maturational trajectory of mean arterial pressure and relative increase in femoral vascular conductance after Picibanil exposure in the present study was not associated with a compensatory increase in fetal heart rate, which may suggest altered baroreflex sensitivity. Previously studies in adult rats exposed to systemic LPS have also reported reduced baroreflex sensitivity.^55^

### Picibanil for treating primary hydrothorax

The present study shows that fetal exposure to even low-dose (0.1 mg) intra-pleural Picibanil is associated with subcortical neuronal loss and diffuse white matter damage. These findings warrant careful assessment of potential neural side-effects of Picibanil use for fetal pleurodesis. Data on long-term behavioural outcomes of children who received Picibanil treatment *in utero* are now being reported. Norgaard et al. reported that 2 out of 14 children treated with Picibanil had learning disabilities by school age.^18^ In another study, 1 out of 9 children treated with Picibanil was reported to develop expressive and receptive language disorder and verbal dyspraxia at one year of age.^14^ The authors acknowledged that adverse effects associated with Picibanil could not be excluded. These were small studies that did not conduct neuroimaging to evaluate any structural differences, emphasizing the need for further large-scale studies. There is, of course, a risk-benefit balance between improved fetal survival and the risk of impaired neurodevelopment. However, at present there is insufficient information on neurodevelopmental outcomes to undertake a balanced risk-benefit analysis. Further, unlike exposure to pathological inflammation, the timing of treatment with Picibanil is known. Therefore, it may be possible to modulate the systemic inflammatory response even after the initial (beneficial) intra-pleural inflammation; for example, by using combined treatment with Picibanil plus an anti-inflammatory agent. Additionally, there is now preclinical and clinical evidence that the maternal gut microbiome may modulate the offspring’s neurodevelopment.^56,57^ Further preclinical studies are needed to examine if modulation of gut microbiota during pregnancy could attenuate the adverse neural effects of Picibanil exposure

## Conclusion

The present study demonstrates that exposure to a single intra-pleural infusion of Picibanil was associated with a diffuse pattern of microglial activation in the white matter, loss of immature and mature oligodendrocytes, subcortical neuronal loss, and associated loss of the maturational increases in both EEG power and arterial blood pressure. The attenuated blood pressure with no change in heart rate infers that there was relative vasodilation due to changes in vascular endothelial function. The precise mechanisms and duration of this effect and the impact on long-term brain development require further longer-term experiments. In addition, higher dose therapy was consistently associated with induction of secondary seizures. These findings should help inform clinical studies testing antenatal Picibanil treatment for fetal hydrothorax/chylothorax.

## Supplemental Material

sj-pdf-1-jcb-10.1177_0271678X231197380 - Supplemental material for The neural and cardiovascular effects of exposure of gram-positive bacterial inflammation in preterm fetal sheep Supplemental material, sj-pdf-1-jcb-10.1177_0271678X231197380 for The neural and cardiovascular effects of exposure of gram-positive bacterial inflammation in preterm fetal sheep by Simerdeep K Dhillon, Christopher A Lear, Joanne O Davidson, Shoichi Magawa, Alistair J Gunn and Laura Bennet in Journal of Cerebral Blood Flow & Metabolism

## Data Availability

The datasets used and/or analysed during the current study are available from the corresponding author on reasonable request.
