# Supplementary material for: The neural and cardiovascular effects of exposure of gram-positive bacterial inflammation in preterm fetal sheep
Source: J Cereb Blood Flow Metab. 2023 Oct 12;44(6):955–69. doi: 10.1177/0271678X231197380 (PMC11318397; doi:10.1177/0271678X231197380)
Supplement: sj-pdf-1-jcb-10.1177_0271678X231197380 - Supplemental material for The neural and cardiovascular effects of exposure of gram-positive bacterial inflammation in preterm fetal sheep [file sj-pdf-1-jcb-10.1177_0271678X231197380.pdf]

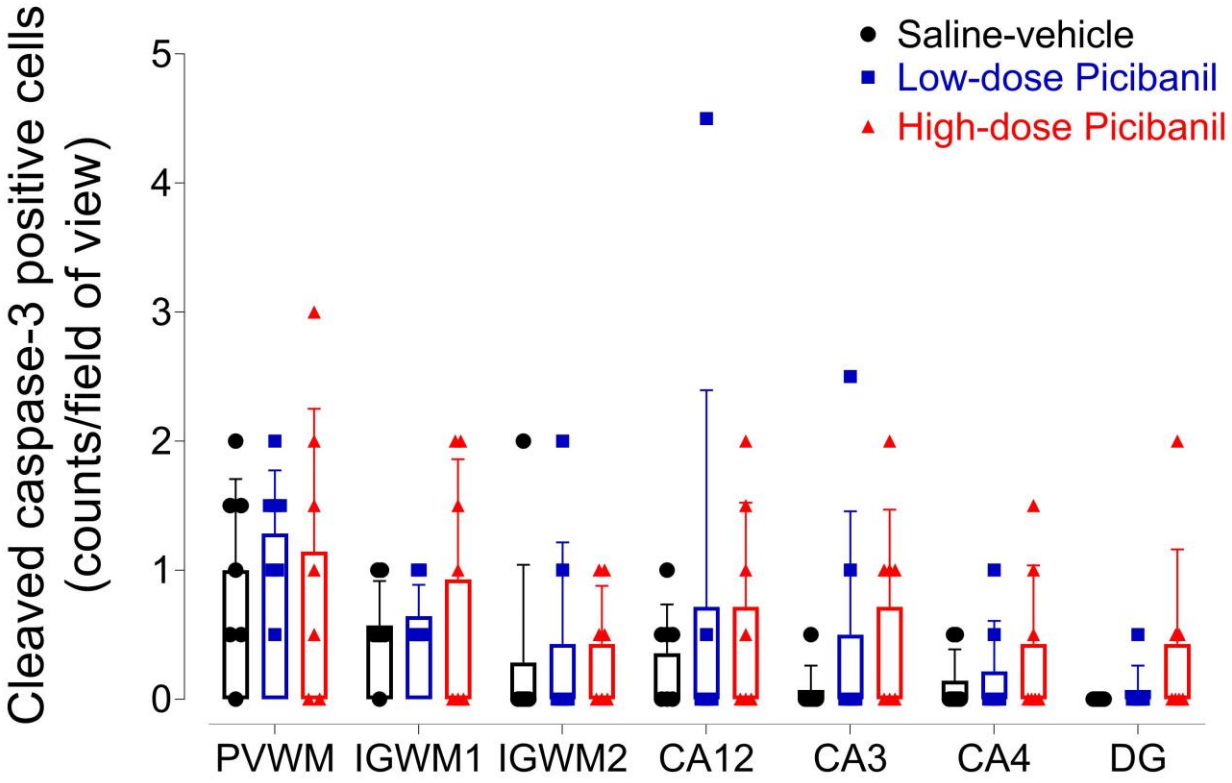

Supplementary Figure 1: Cell density of Cleaved caspase-3 positive apoptotic cells in the periventricular and parasagittal intragyral white matter areas, and CA12, CA3, CA4, and DG of the hippocampus in the saline-vehicle (closed black circles, n=7), low-dose Picibanil (closed blue squares, n=7) and high-dose Picibanil (red triangles, n=7) groups at one week after intrapleural Picibanil infusion. Data are presented as individual animals (the bars show the mean and SD), and were assessed using mixed-design ANOVA with regions as repeated measures and experimental groups as independent variables. Comparisons between the groups were performed using Tukey's post hoc test. CA: cornu ammonis, DG: dentate gyrus.
